# A Set of Highly Polymorphic Microsatellite Markers for Genetic Diversity Studies in the Genus *Origanum*

**DOI:** 10.3390/plants12040824

**Published:** 2023-02-12

**Authors:** Marina Alekseeva, Mila Rusanova, Krasimir Rusanov, Ivan Atanassov

**Affiliations:** Department of Agrobiotechnology, AgroBioInstitute, Agricultural Academy, 1164 Sofia, Bulgaria

**Keywords:** microsatellite markers, SSR, *Origanum*, Greek oregano, common oregano, sweet marjoram, genetic diversity

## Abstract

This study reports the development of a set of 20 highly polymorphic genomic SSR markers which can be used for both cultivar identification and genetic diversity studies in several *Origanum* species, including some of the most popular ones like Greek oregano (*Origanum vulgare* L. ssp. *hirtum*), common oregano (*O. vulgare* L. ssp. *vulgare*), and sweet marjoram (*O. majorana* L.). Analysis of the polymorphic information content (PIC) showed an average PIC value of 0.75 with a minimum of 0.41 and a maximum of 0.89, where 17 of the markers showed PIC values above 0.73. Comparative analysis of the genetic diversity of eight natural populations of Greek oregano in Bulgaria showed that six of the genomic SSR markers revealed significantly higher portions of genetic diversity in the populations, compared to 12 EST SSR markers used in our previous study. We also compared the performance of the same six genomic SSR markers with the results for eight SRAP primer combinations, which showed that SRAP markers captured more precisely the genetic structure in natural populations. The developed highly polymorphic genomic SSR markers can be successfully applied to evaluation of the genetic diversity in the genus *Origanum,* based on the expected and observed heterozygosity in the populations as well as for easy identification of breeding lines and cultivars based on unique SSR fingerprints.

## 1. Introduction

The members of the genus *Origanum* (Lamiaceae), which includes more than 50 species and a number of subspecies, have been known since ancient times both as culinary spices and as herbs with medicinal properties. Natural populations of the different species and subspecies of the genus are widespread throughout geographic regions spanning the Mediterranean, western and southwestern Eurasia, and the Irano-Turanian region [1]. Even more, *Origanum* species have been cultivated on continents where they are not native, and recently New Zealand has claimed to be one of the biggest producers of oregano in the world [2].

Some of the most popular members of the genus include Greek oregano (*Origanum vulgare* ssp. *hirtum*) with its high essential oil content, common oregano (*O. vulgare* L. ssp. *vulgare*), and sweet marjoram (*O. majorana* L.). Numerous studies over the years have shown that *Origanum* species exhibit diverse biological activities, mainly due to the essential oil accumulated in the aerial parts of the plants, including antioxidant activity, antibacterial activity, cytotoxic and anti-proliferative activities against human cancer cells, anti-inflammatory activity, antidiabetic activity, and antiparasitic activity, etc. [1,3]. The great interest in these species and their significant economic value has led to the need to establish collections of genetic resources reflecting the diversity in natural populations (both genetic and metabolic) and to develop high performing elite cultivars [4,5].

To date, genetic diversity studies in *Origanum* species have mainly been carried out using dominant types of PCR-based molecular markers like AFLP, RAPD, SRAP, and ISSR [1]. Although such PCR-based dominant markers are easy to apply since they do not require previous knowledge of the sequence of the studied loci, they provide limited information regarding the allelic state of the loci. Moreover, data generated by dominant markers is often difficult to reproduce in various laboratories, as a number of factors can affect the PCR-based banding pattern due to the non-locus-specific character of the primers used. All this significantly hampers identification of breeding lines and cultivars in the genus *Origanum* using DNA markers as well as implementation of marker-assisted selection (MAS) in breeding programs.

Microsatellite markers, also known as simple sequence repeats (SSRs), are PCR-based co-dominant type of molecular markers that have been the preferred marker system for genotype identification in different plant species [6]. The protocols for SSR analysis and the generated data are easily transferable between laboratories and are highly reproducible. The resulting SSR profiles can be readily organized in online databases and used for analysis of genetic authenticity and cultivar identification [7]. So far, the only SSR markers developed in the genus *Origanum* have been based on expressed sequence tags (ESTs), resulting in low polymorphic information content (PIC) and, respectively, low applicability in genetic diversity studies [4,8].

Here we report the development of a set of highly polymorphic genomic SSR markers based on NGS sequencing of genomic DNA from *O. vulgare* ssp. *hirtum*. We tested the developed markers on a small set of genetically diverse *O. vulgare* ssp. *hirtum* lines previously characterized with SRAP markers, and we reported the transferability of the developed SSR set to *O. vulgare* ssp. *vulgare* and *O. majorana* L. Furthermore, we compare and discuss the performance of the developed genomic SSRs with SRAP markers previously used for analysis of the genetic diversity and genetic structure of natural populations of *O. vulgare* ssp. *hirtum* in Bulgaria.

## 2. Results and Discussion

Twenty-six SSR markers derived from *O. vulgare* ssp. *hirtum* were used for evaluation of the level of polymorphism through analysis of a set of 10 plants, eight of which represent Greek oregano (*O. vulgare* ssp. *hirtum*), one, common oregano (*O. vulgare* ssp. *vulgare*) and one, sweet marjoram plant (*O. majorana* L). Twenty of the SSR primers (Table 1) showed diversity among the analyzed samples. Eighteen of the primer pairs successfully amplified genomic DNA from common oregano (*O. vulgare* ssp. *vulgare*), and 16 SSR markers were fully transferable among Greek oregano, common oregano and sweet marjoram (Table 1). Table 1 shows the calculated PIC values for each primer pair. 

The number of different alleles identified for each locus varied between five and 13, corresponding to an average PIC value of 0.75 with a minimum of 0.41 and maximum of 0.89. It is noteworthy that 17 of the markers showed PIC values above 0.73, demonstrating that the selected set of SSR markers were highly informative. In general, SSR markers with PIC values above 0.5 are considered highly informative and suitable for genetic diversity studies [9]. In comparison, Alekseeva et al. used a set of 12 EST derived SSR markers [8] to assess the genetic diversity in eight natural populations of *O. vulgare* ssp. *hirtum* from two regions in Bulgaria [4]. The data showed that the average PIC value for the EST derived SSR markers was 0.48 (unpublished PIC calculation data from Alekseeva et al., 2021) with a minimum of 0.29 and a maximum of 0.71. Furthermore, eight of the EST SSRs had PIC values below 0.5. The present data further supports the higher PIC values of genomic SSRs compared to EST derived SSR markers, also observed in other studies, due to the lower extent of toleration of the mutations located in gene coding sequences associated with the ESTs [10,11]. The low PIC values of the EST-SSR markers used by Alekseeva et al., 2021 resulted in prevention of differentiation among the studied populations and even the regions of the analyzed *O. vulgare* ssp. *hirtum* natural populations [4]. To further clarify the efficiency and informativeness of the studied SSR markers, we used a subset of six highly polymorphic SSRs with PIC values above 0.77 (R-38C, R-40C, R-103C, R-105C, R-115C, and R-6M) to evaluate the genetic diversity and structure of eight natural populations of *O. vulgare* ssp. *hirtum*, analyzed previously by Alekseeva et al., 2021, using SRAP markers [4], as well as to carry out a phylogenetic analysis of plants from a single population of this species. We used a set of 96 genomic DNA samples analyzed by Alekseeva et al., 2021 corresponding to plants from eight different populations, including two from the Kresna Gorge region (pops 1–2) and six from the Eastern Rhodopes region (pops 3–8) in Bulgaria. Comparison of the genetic diversity parameters using genomic SSR and EST-SSR markers revealed several distinct differences. The mean of both the number of different alleles and the effective number of alleles based on genomic SSR markers data was almost twice as high compared to EST-SSR markers (Table 2). The calculated expected heterozygosity based on genomic SSR markers was 0.711, indicating high genetic diversity in the studied populations. At the same time, the expected heterozygosity based on EST-SSR markers was below 0.5, thus hiding a large portion of the actual diversity in the populations. Correspondingly, Shannon’s diversity index calculated from genomic SSR data was significantly higher than the value derived from the EST-SSR data. For both types of markers, the fixation index was close to 0 as expected for random mating in the natural populations. The overall comparison of genomic SSR and EST-SSR data clearly demonstrates that the EST-SSR derived data significantly underestimate the actual genetic diversity in the studied populations and should be used with caution.

**Figure 1:**
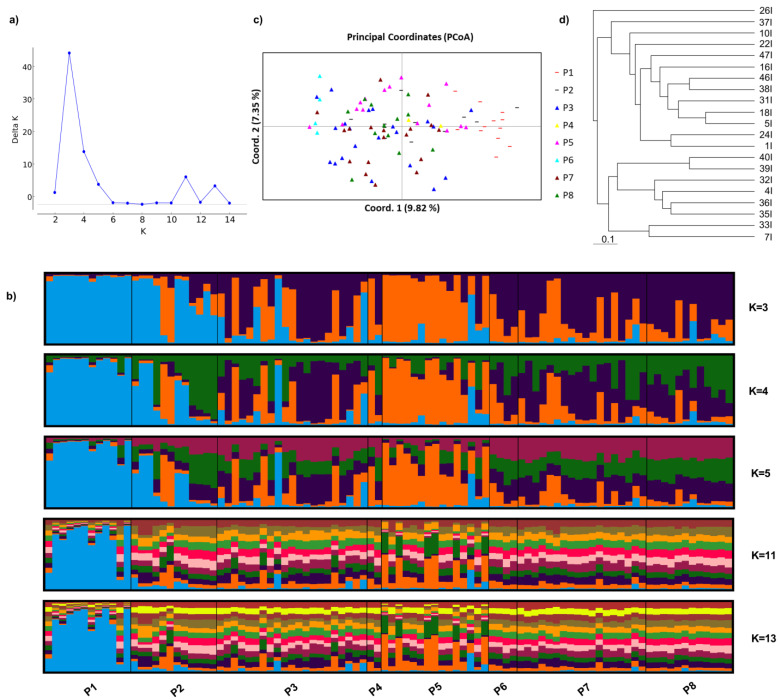
Analysis of the genetic structure of 8 natural populations of *Origanum vulgare* subsp. *hirtum* (Link) Ietsw in Bulgaria using 6 highly polymorphic genomic SSR markers. (**a**) Determining the most probable number of genetic clusters using the Delta K method by Evanno et al. [12]; (**b**) Bar plot representing the genetic structure of the studied populations at several K values; (**c**) Principal Coordinate Analysis based on genomic SSR marker data for the analyzed 8 populations. Samples marked with “-” belong to the Kresna Gorge region while samples marked with “△” belong to the Eastern Rhodopes region; (**d**) Phylogenetic tree constructed by the Fitch-Margoliash method representing the genetic similarities among the plants in population 3.

We further analyzed the genetic structure of the populations with the same set of six genomic SSR markers and compared the results with those obtained with SRAP markers from the previous study by Alekseeva et al., 2021 [4]. In order to determine the most probable number of genetic clusters, we used the Delta K method developed by Evanno et al., which showed a maximum Delta K value at K = 3 with additional peaks at K = 4, 5, 11, and 13 (Figure 1a). Visualization of the genetic structure at all mentioned K values (Figure 1b) showed no significant genetic structuring above K = 5. Detailed analysis of the genetic structure at K = 3 to K = 5 showed that population 1 from the Kresna Gorge region was clearly differentiated from all populations from the Eastern Rhodopes region, as was previously demonstrated by Alekseeva et al., 2021 using a set of 8 SRAP primer combinations. Similarly, population 5 from the Eastern Rhodopes region was also highly differentiated from the rest of the populations from the Eastern Rhodopes region as was also reported by Alekseeva et al., 2021. However, population 2 from the Kresna Gorge region was only partially differentiated from the Eastern Rhodopes region in the current study, while Alekseeva et al., 2021 were able to clearly differentiate between this population and all populations from the Eastern Rhodopes region. Additionally, the performed Principal Coordinate Analysis (PCoA) (Figure 1c) confirmed the lack of clear separation between the two studied regions where samples from population 2 (Kresna Gorge region) were scattered among samples from the Eastern Rhodopes region. Overall, the analysis of 96 samples from eight natural populations of *O. vulgare* L. ssp. *hirtum* with six highly polymorphic SSR markers developed in the current study was only partially able to differentiate between the 8 studied natural populations. Overall, the results showed that in terms of studying the genetic structure of natural populations in our study, SRAP markers analyzed on a capillary sequencer were superior, probably due to the large number of analyzed loci with only a few SRAP primer combinations. In this case, the use of a greater number of SSR markers would be needed to match the resolving power of SRAP markers.

We further used the subset of genomic SSR marker data for the plants from population 3 to perform clustering analysis using the proportion of shared alleles for calculating the genetic distance between the analyzed plants and phylogenetic tree construction by the Fitch-Margoliash method. The performed clustering aimed to simulate analysis of genetic diversity in a collection of genetic resources where members of the collection are typically diverse in terms of their genetic structure and classical phylogenetic approaches including building a phylogenetic tree are often employed. The obtained phylogenetic tree (Figure 1d) demonstrates the high-resolution power of the applied set of genomic SSR markers, capable of distinguishing among all analyzed plants from the population. The dendrogram also showed clustering of the samples which reflected well the color pattern of the samples from population 3 in the genetic structure analysis (Figure S1). The calculated probability of identity (PI) for the SSR markers were 0.21 (R-40C), 0.17 (R-6M), 0.1 (R-103C), 0.13 (R-38C), 0.06 (R-105C) and 0.12 (R-115C) with a total PI of 3.126384 x 10^-6^. The relatively low value of total PI indicated that the set of SSR markers used is suitable for identification of individual plants within the population. The results of the phylogenetic analysis also suggest that the selected genomic SSR markers can be effectively applied for characterization of genetic resources collections of oregano species and implementation in genetic diversity studies applying classical phylogenetic approaches.

Taken together, the results presented above demonstrate that the genomic SSR markers developed in the current study are highly informative and can be effectively applied both to characterize the genetic diversity of *O. vulgare* ssp. *hirtum* populations and other oregano species, as well as to characterize oregano genetic resources collections. Although the SSR markers were less effective than SRAP markers in differentiating the studied populations in our study, it should not be generalized that genomic SSRs are less effective than SRAPs for studying the genetic structure of natural populations. Additionally, the co-dominant nature of SSR markers makes them a valuable tool for in-depth evaluation of the genetic diversity in populations providing data on the expected and observed heterozygosity, as well as for evaluation of the heterozygosity deficit. The results of the application of genomic SSR markers for genotyping and phylogenetic analysis suggest that they can be effectively applied for characterization of genetic resources collections, where the developed unique SSR allele patterns can be further used for identification of breeding lines and cultivars, construction of databases, analysis of genetic authenticity, and homogeneity of planting material.

## 3. Materials and Methods

### 3.1. Plant Material, Genomic DNA Isolation and DNA Samples

The plants used for purification of genomic DNA were grown in greenhouse conditions in the experimental station of ABI in the town of Kostinbrod. Eight Greek oregano (*O. vulgare* ssp. *hirtum*) plants were selected based on their belonging to different genetic clusters in a previous analysis with SRAP markers of seed derived plants from natural populations (unpublished data). *O. vulgare* ssp. *vulgare* and *O. majorana* L. were grown from seeds purchased from Agrara Ltd. and Florian Ltd., Bulgaria. A single plant from each *O. vulgare* ssp. *vulgare* and *O. majorana* L. was used for genomic DNA isolation. Genomic DNA was isolated from leaves which were immediately frozen after removal from the plants in a plastic container. The plant material was ground to fine powder using a TissueLyser (Qiagen) laboratory mill. Genomic DNA was purified according to the CTAB protocol [13]. All prepared DNA samples were diluted to a final concentration of 25 ng/µL in ultrapure water. Additionally, a set of 96 genomic DNA samples from plants from eight different natural populations previously analyzed by SRAP markers in our previous study [4] was used for comparative analysis with the genomic SSR markers tested in the present study. The geographic coordinates of these *O. vulgare* ssp. *hirtum* natural populations, as well as geographic maps with their locations marked, were presented in Alekseeva et al., 2021 [4].

### 3.2. Identification of SSR Sequences Based on NGS

Microsatellite sequences from *O. vulgare* L. ssp. *hirtum* were identified as a service by Ecogenics GmbH following NGS sequencing of an *O. vulgare* L. ssp. *hirtum* genomic DNA sample. The Illumina TruSeq nano DNA library was sequenced on an Illumina MiSeq sequencing platform using a nano v2 500 cycles sequencing chip. The resulting paired-end reads which passed Illumina’s chastity filter were subjected to de-multiplexing and trimming of Illumina adaptor residuals. Subsequently the quality of the surviving reads was checked with FastQC v0.11.8 [14]. In a next step, the paired-end reads were quality filtered and merged with USEARCH v11.0.667 [15] to in silico reform the sequenced molecules. The resulting merged reads were screened with the software Tandem Repeats Finder, v4.09 [16]. After this process, 6121 merged reads contained a microsatellite insert with a tetra- or a trinucleotide of at least six repeat units or a dinucleotide of at least ten repeat units. Primer design was performed with primer 3 [17,18]. Raw NGS sequences can be accessed at the NCBI Sequence Read Archive under project number PRJNA921701.

### 3.3. PCR Amplification and Analysis of SSR Markers

One hundred nineteen primers corresponding to SSR loci identified by NGS sequencing of gDNA of *O. vulgare* L. ssp. *hirtum* were tested for PCR amplification of gDNA from the same sample used for NGS sequencing. Two different types of tails were added at the 5′ end to each forward primer based on the calculated melting temperature of the respective reverse primer using Primer 3 Plus [18]. The tails used were Tail M13 (5′-GTAAAACGACGGCCAGT-3′) and Tail C (5′-CAGGACCAGGCTACCGTG-3′) [19,20]. Tail C was used when the Tm of the reverse primer was > 58 °C. Tail M13 was used when the Tm of the reverse primer was ≤58 °C. Based on the specific tail used, the annealing temperatures of the PCR reactions were 57 °C for Tail C and 54 °C for Tail M13. The PCR reactions were performed in a volume of 16 µL, containing 0.8 µL of Fw primer (3 pmol/µL), 1 µL of Tail primer (10 pmol/µL) labelled with FAM (Table 1), 1 µL of Rev primer (10 pmol/µL), 8 µL 2x MyTaq^TM^ Mix (Bioline), 4 µL ultra-pure water, and 1.3 µL gDNA, using the following PCR conditions: 95 °C for 3 min followed by 33 cycles of 95 °C for 15 s, T annealing (Table 1) for 30 s, 72 °C for 30 s and final elongation at 72 °C for 10 min. Fragment analysis was performed on the ABI 3130 Genetic Analyzer (Thermo Fisher Scientific, Waltham, MA, USA) using 36-cm long capillaries (Thermo Fisher Scientific, Waltham, MA, USA), Pop-7 polymer (Thermo Fisher Scientific, Waltham, MA, USA), and GeneScan^TM^ 500 LIZ^TM^ as a size standard (Thermo Fisher Scientific, Waltham, MA, USA). GeneMapper 4.0 (Thermo Fisher Scientific, Waltham, MA, USA) was used for fragment sizing, and alleles were reported as base pairs after subtracting the length of the respective tail added to the Fw primer. The resulting capillary sequencer electropherograms were visually inspected for clarity and number of obtained fragments. SSR primer combinations where multiple PCR products were observed were excluded from further analysis resulting in a total of 26 SSR markers selected for analysis in the study.

### 3.4. Statistical Analysis

The polymorphic information content (PIC) of SSR markers was calculated using PowerMarker [21]. GenAlEx 6.5 was used for calculating genetic diversity parameters including number of alleles, number of effective alleles, Shannon’s diversity index, expected heterozygosity, observed heterozygosity, and fixation index as well as for performing Principal Coordinate Analysis [22]. Identity 1.0 was used for calculating probability of identity of SSR markers [23]. Phylogenetic tree was constructed using Microsat [24] for calculation of genetic distances based on proportion of shared alleles and KITSCH from the PHYLIP package [25] for building the tree. Visualization of the tree was done using Treeview v 1.6.6 [26]. The genetic structure of the tested populations was analyzed using Structure 2.3.4 [27] where Admixture was used as an ancestry model, Length of Burnin Period was set to 100,000, and the Number of MCMC Reps after Burnin was set to 200,000. The number of presumed clusters (K) was set from 1 to 15, and 10 iterations were performed for each K value. Parallelization of Structure 2.3.4 calculations was achieved using EasyParallel [28]. The most probable K was determined using the method by Evanno et al. [12] with the help of Structure Harvester [29]. The different iterations at a single K value were combined using Clumpak [30].

## 4. Conclusions

A set of 20 highly informative genomic SSR markers from *O. vulgare* L. ssp. *hirtum* was developed, 16 of which can be successfully transferred between species in the genus *Origanum,* including Greek oregano (*O. vulgare* L. ssp. *hirtum*), common oregano (*O. vulgare* L. ssp. *vulgare*), and sweet marjoram (*O. majorana* L.). Our results clearly demonstrate that the developed genomic SSR markers are able to capture a significantly higher portion of the genetic diversity in the natural populations of Greek oregano compared to EST-SSR markers. Although in our comparative study SRAP markers were superior to genomic SSR markers for studying the genetic structure and differentiation of natural *Origanum* populations, the highly developed polymorphic genomic SSR markers can be successfully applied for in-depth evaluation of the population genetic diversity including the expected and observed heterozygosity. The application of a small subset of six genomic SSR markers for phylogenetic analysis of plants from a single population suggests it can be effectively applied for characterization of *Origanum* genetic resources collections as well as for identification of breeding lines and varieties.

## Figures and Tables

**Table 1:** SSR primer pairs used for genetic diversity analysis in the current study. Bold letters indicate tails added to the Fw primer (tail “M13” 5′-GTAAAACGACGGCCAGT-3′ and tail “C” 5′-CAGGACCAGGCTACCGTG-3′). Ta—annealing temperature, PIC—polymorphic information content, GO—Greek oregano (*Origanum vulgare* ssp. *hirtum*), CO—common oregano (*Origanum vulgare* ssp. *vulgare*), M—sweet marjoram (*Origanum majorana* L.).

SSR Locus	Repeat Motif		Primers	Ta, °C	Amplicon Size Range (bp)	PIC	Successful PCR Amplification of
R-6M	(TTG)_16_	FW	5′-**GTAAAACGACGGCCAGT**CTGTCGATGCCACTTCTTCAC-3′	54	192–229	0.77	GO, CO, M
		Rev	5′-TCAGGTGAAGCTACTACCCAC-3′				
R-12M	(TAAA)_7_	FW	5′-**GTAAAACGACGGCCAGT**AGTCCTAAGCTACATTTGATATTGCC-3′	54	226–234	0.41	GO, CO, M
		Rev	5′-ACTGCGATAATTAGTGGTAGGTG-3′				
R-22M	(CT)_12_	FW	5′-**GTAAAACGACGGCCAGT**GCTTCTTGATTTTTAGCTTTCATTGTC-3′	54	142–168	0.81	GO, CO, M
		Rev	5′-GTTGACTTCCACATCAACAGTAAG-3′				
R-29C	(AG)_12_	FW	5′-**CAGGACCAGGCTACCGTG**GGGTAGCAGGGTTGATTTCC-3′	57	92–128	0.74	GO
		Rev	5′-ACGGAGGTGCTCACCATAAG-3′				
R-38C	(CT)_17_	FW	5′-**CAGGACCAGGCTACCGTG**AATATTTTCAGCCGACTCTTCG-3′	57	93–123	0.8	GO, CO, M
		Rev	5′-CCGTCACGCTTACCTTTTGG-3′				
R-39C	(CA)_13_	FW	5′-**CAGGACCAGGCTACCGTG**AAGACCATTCTGTGGGGGAC-3′	57	124–146	0.73	GO, CO, M
		Rev	5′-TGCATGCGCCATCATAAGAC-3′				
R-40C	(ATCT)_9_	FW	5′-**CAGGACCAGGCTACCGTG**AACTTTAGACACGGATGCGG-3′	57	103–139	0.87	GO, CO, M
		Rev	5′-TGCATTTGCACGTAACTTTCTAC-3′				
R-57C	(CT)_15_	FW	5′-**CAGGACCAGGCTACCGTG**ACCTTCACCGTTGTTAGGGG-3′	57	137–153	0.77	GO, CO, M
		Rev	5′-AACGGTATCGAGAGTGTGCG-3′				
R-75C	(GA)_12_	FW	5′-**CAGGACCAGGCTACCGTG**GCGTACCAGTTTCCTGGATG-3′	57	161–173	0.52	GO, CO, M
		Rev	5′-CTGCGGACGAAGCATAACTC-3′				
R-77C	(TCA)_11_	FW	5′-**CAGGACCAGGCTACCGTG**ACAACTGTTCCAAGAATCAGAGC-3′	57	170–210	0.75	GO, CO, M
		Rev	5′-CCCCTGTAAGTAGCAATCGTC-3′				
R-81C	(AT)_23_	FW	5′-**CAGGACCAGGCTACCGTG**TCTCCGATAAACAGGGGAGC-3′	57	137–199	0.74	GO, CO
		Rev	5′-ACGAAGTCATTTCTTTTAATCTTGC-3′				
R-83C	(AG)_17_	FW	5′-**CAGGACCAGGCTACCGTG**AGGGCCGAGCACTTAAATAAC-3′	57	173–215	0.73	GO, CO, M
		Rev	5′-AATTGAAGGCTATGACCGGC-3′				
R-85C	(AT)_11_	FW	5′-**CAGGACCAGGCTACCGTG**TCGCAGGCAGGTTGATAGAG-3′	57	165–241	0.81	GO, CO, M
		Rev	5′-TGATGGTGTTCTTTTCAGCTCG-3′				
R-88C	(CA)_19_	Fw	5′-**CAGGACCAGGCTACCGTG**TCAAAGTCCGAAAACAGTTCTAAATC-3′	57	175–257	0.89	GO, CO, M
		Rev	5′-CGTTCCAAGCAATAGCCTCC-3′				
R-94C	(GA)_13_	FW	5′-**CAGGACCAGGCTACCGTG**TGCAGAGTGATAAGCTCGTTAG-3′	57	196–224	0.75	GO
		Rev	5′-GTCAAGACCCATAACTCGTGTC-3′				
R-103C	(GA)_13_	FW	5′-**CAGGACCAGGCTACCGTG**AAAAGGCGGCTGCTGATTAC-3′	57	199–221	0.84	GO, CO, M
		Rev	5′-CCCAAGTTCTTGCGAACAGG-3′				
R-105C	(GA)_17_	FW	5′-**CAGGACCAGGCTACCGTG**TTGGAGGCTTACTGTCTGGG-3′	57	207–273	0.86	GO, CO, M
		Rev	5′-ATGTTGGGAGCTTTCATGGC-3′				
R-114C	(AG)_11_	FW	5′-**CAGGACCAGGCTACCGTG**ACCAGAAATGGCCTCTACCG-3′	57	226–236	0.64	GO, CO, M
		Rev	5′-GTCCGACAATCACTTGCTCC-3′				
R-115C	(AG)_15_	FW	5′-**CAGGACCAGGCTACCGTG**CCATGGCTTCCGATTTGAGC-3′	57	221–253	0.8	GO, CO, M
		Rev	5′-GCAAATTAATCAAACGGTAAACTGTC-3′				
R-116C	(TTAA)_8_	FW	5′-**CAGGACCAGGCTACCGTG**TCGTAACATCCCTCGTTGAC-3′	57	210–260	0.77	GO, CO
		Rev	5′-CCGTGAAGCACAGGATTTGG-3′				

**Table 2:** Genetic diversity parameters (mean values with standard errors) of the studied 8 populations of *Origanum vulgare* ssp. *hirtum* based on data from 6 genomic SSR markers (current study) and 12 EST SSR markers (unpublished data from Alekseeva et al., 2021). Na = number of different alleles, Ne = effective number of alleles, I = Shannon’s diversity index, Ho = observed heterozygosity, He = expected heterozygosity, Fis = fixation index.

	Na	Ne	I	Ho	He	Fis
**Genomic SSR**	6.604 ± 0.384	4.254 ± 0.241	1.534 ± 0.069	0.745 ± 0.031	0.711 ± 0.024	−0.051 ± 0.032
**EST SSR**	3.818 ± 0.164	2.147 ± 0.077	0.879 ± 0.037	0.448 ± 0.021	0.482 ± 0.018	0.063 ± 0.024

## Data Availability

Raw NGS sequences can be accessed at the NCBI Sequence Read Archive under project number PRJNA921701.

## Supplementary Materials

The following supporting information can be downloaded at: https://www.mdpi.com/article/10.3390/plants12040824/s1, Figure S1: Genetic structure analysis (a) and phylogenetic tree (b) of population 3. Same symbol below sample names in the genetic structure plot as well as next to the samples names in the phylogenetic tree indicate samples with similar color pattern in the genetic structure plot belonging to the same clusters in the phylogenetic tree.
