# Supplementary figures and images for: A Set of Highly Polymorphic Microsatellite Markers for Genetic Diversity Studies in the Genus Origanum
Source: Plants (Basel). 2023 Feb 12;12(4):824. doi: 10.3390/plants12040824 (PMC9965030; doi:10.3390/plants12040824)

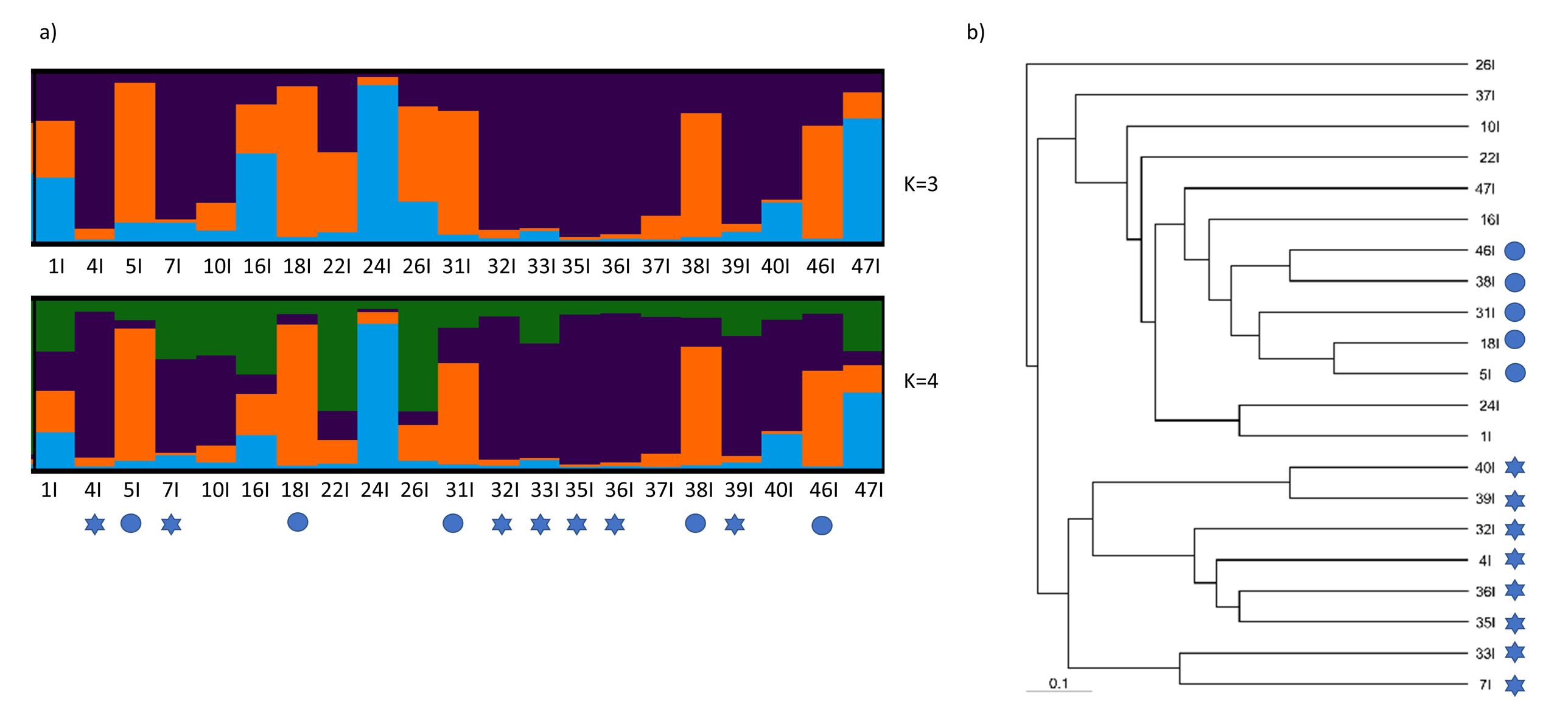

Supplement: Supplementary file 1 [file plants-12-00824-s001.zip › plants-2147469-supplementary.jpg]
